# Surface Modification of Silica Particles with Adhesive Functional Groups or Their Coating with Chitosan to Improve the Retention of Toothpastes in the Mouth

**DOI:** 10.1021/acs.langmuir.2c03269

**Published:** 2023-01-17

**Authors:** Sam R. Aspinall, Vitaliy V. Khutoryanskiy

**Affiliations:** † Department of Pharmacy & Research Centre in Topical Drug Delivery and Toxicology, University of Hertfordshire, HatfieldAL10 9AB, Hertfordshire, U.K.; ‡ Department of Pharmacy, University of Reading, Whiteknights, PO Box 224, ReadingRG6 6DX, U.K.

## Abstract

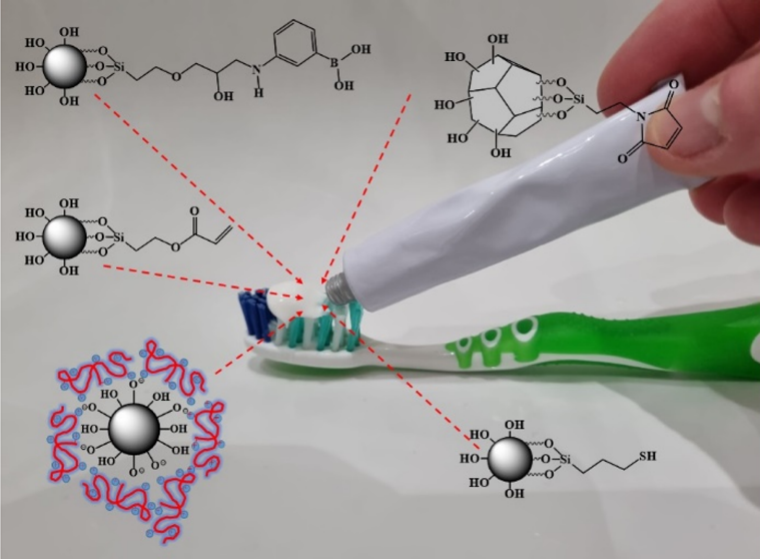

Silica is widely used in the oral care formulations to act as an abrasive and to give the products its distinct physical properties. In this study, silica particles were synthesized using a co-condensation of tetraethyl orthosilicate with a series of functional silane compounds [(3-mercaptopropyl)trimethoxysilane, (3-glycidyloxypropyl)trimethoxysilane, and (3-acryloxypropyl)trimethoxysilane)]. The surface of the particles based on tetraethyl orthosilicate and (3-glycidyloxypropyl)trimethoxysilane was then further modified with 3-aminophenylboronic acid. Commercial Aerosil R972 Pharma silica particles were also coated with chitosan. Additionally, commercially available (3-maleimido)propyl-functionalized silica particles were used in this study. All these functionalized silica particles were incorporated into toothpaste formulations, and their retentive properties were tested on *ex vivo* sheep tongue mucosa models using fluorescent microscopy-based flow-through techniques. Those surfaces with chitosan, phenylboronic acid, and acryloyl groups were shown to provide a significant improvement in the retention of the oral care formulations during the retention testing. The retention of toothpastes containing silica functionalized with maleimide and thiol groups was also superior compared to that of unmodified silica particles. This study synthesized and tested a range of silica particles and demonstrated that the functionalized silica incorporated into toothpastes can significantly improve the retention of these formulations on oral mucosal surfaces.

## Introduction

Maintaining good oral health is critical to the wellbeing of all humans, with diets rich in sugars and acidic foods/drinks becoming increasingly available and longer life expectancies, the need to retain, maintain, and protect our teeth and gums is more important than ever before. Poor oral health can lead to dental caries, gum diseases, and tooth loss, potentially leading to cardiovascular disease, cancer, and strokes.^1,2^ To prevent this, oral care products have continued to develop from simple powders and pastes to complex multifunctional formulations, capable of cleaning and protecting teeth throughout the day.

One of the major limitations of toothpastes and oral care formulations is their poor retention in the mouth as most of the toothpaste is quickly eliminated post brushing. The active ingredients from these formulations are quickly washed away, reducing their effectiveness. The constant diffusion gradient created by good blood flow and large surface area, deglutition, mucus secretions, and mastication and consumption of food quickly remove any remaining formulation, or its components applied to the oral cavity.^3,4^

One way of increasing the retention time of oral care products in the mouth is through the incorporation of mucoadhesives into the formulation. Mucoadhesion is described as the attractive interaction between a pharmaceutical dosage form and a mucosal membrane at their interface.^5−8^ The addition of polymers that exhibit strong mucoadhesive properties has been shown to increase the retention time of oral care formulations on mucosal surfaces.^9^ Although these polymers were used in only small quantities relative to the formulation, they were able to significantly increase the toothpaste’s retention time.

When looking to further improve oral care formulations, one approach to extending the retention time of formulations at mucosal surfaces is by functionalizing silica with mucoadhesion-enhancing functional groups or polymers. Silica is commonly used as an abrasive and/or a thickening agent in cosmetic and pharmaceutical formulations. The particles incorporated into these formulations typically range from 4 to 12 μm.^10^ Some of the advances in the use of functionalized silica for transmucosal drug delivery were recently reviewed by Ways *et al.*;^11^ however, the use of mucoadhesive silica particles is still insufficiently exploited in pharmaceutical formulations. In toothpastes, silica can make up to 40% of a formulation.^12^ The silica plays an active role in removing the bacteria and stains from the mucosal and enamel surfaces while also playing a major role in the formulation’s rheological properties. The large percentage used in toothpastes as well as versatility, biocompatibility, and ability to be chemically modified with a variety of groups make it an ideal target for functionalization with the aim of improving the formulations retention on mucosal surfaces.

Functionalized silica particles can be prepared using several approaches. One of the approaches is the hydrolysis and condensation of tetraethoxysilane (TEOS) to form silica particles, whose surface is rich in hydroxyl groups. This approach developed in 1968 is called Stöber process.^13^ These particles can be subsequently post-functionalized by reactions with various silane coupling agents leading to the introduction of various functional groups on the surface. Examples of this post-functionalization include reactions with (3-mercaptopropyl)trimethoxysilane (MPTS)^14^ or (3-aminopropyl)trimethoxysilane.^15,16^ Alternatively, functionalized silica particles can be prepared by a one-step synthesis, which involves self- or co-condensation of MPTS and other mercapto-silanes^17,18^ and/or (3-aminopropyl)trimethoxysilane.^16,19^

The introduction of functional groups to silica’s surface can potentially increase its mucoadhesive properties leading to longer retention of the particles in the oral cavity. Alternatively, the retention properties of silica particles could be improved by the addition of mucoadhesive polymers or monomers. Several types of functional groups such as thiols, acryloyls, maleimides, and phenylboronic acid could potentially improve the mucoadhesive properties due to their ability to form covalent links with mucins and other biological tissues.^20^ Thiol groups can form disulfide bridges with cysteine-rich subdomains of mucus glycoproteins *via* exchange reactions or through oxidation.^21−23^ Acryloyl and maleimide groups can form covalent bonds with thiol groups present in mucins *via* a Michael-type addition reaction.^24−26^ The two hydroxyl groups on phenylboronic acid (PBA) allow it to form dynamic covalent bonds with 1,2-cis-diols on mucin molecules *via* a condensation reaction making it a very strong and stable mucoadhesive.^27−29^ Chitosan is an aminopolysaccharide derived from chitin by its partial deacetylation and known for being one of the strongest mucoadhesive polymers.^8^ The mucoadhesive properties of chitosan are due to the electrostatic interactions, hydrogen bonding, and hydrophobic effect with the negatively charged mucosa.^30−32^ The mucoadhesive properties of these particles will improve the retention of the pharmaceutical formulation they are imbedded in, especially if in large quantities like oral care products. Once modified, the silica particles have the potential to be loaded up with antimicrobial, anti-inflammatory, or desired active pharmaceutical ingredients.^33−35^

In this work, the mucoadhesive silica particles were made through the co-condensation of TEOS with MPTS and (3-acryloxypropyl)trimethoxysilane (APTS). The silica particles were additionally prepared by co-condensation of TEOS with 3-glycidyloxypropyl)trimethoxysilane (GOPS), and then their surface was subsequently modified by the reaction with 3-aminophenylboronic acid (APBA). Additionally, commercially available (3-maleimido)propyl-functionalized silica and Aerosil R972 silica particles were used. The surface of Aerosil R972 silica was modified by coating with chitosan. All these particles were characterized using scanning electron microscopy (SEM), transmission electron microscopy (TEM), and thermal gravimetric analysis (TGA). Then these mucoadhesive silica particles were incorporated into toothpastes, and their retention was tested on the sheep tongue tissues *ex vivo*. To the best of our knowledge, this is the first study demonstrating the use of silica particles functionalized with various mucoadhesive groups and chitosan to improve the retention properties of toothpastes.

## Experimental Section

### Materials

3-Mercaptopropyltrimethoxysilane (95%), tetraethyl orthosilicate (98%), (3-glycidyloxypropyl)trimethoxysilane (98%), chitosan (low molecular weight), 3-aminophenylboronic acid, (3-maleimido)propyl-functionalized silica gel, ethanol (99%), fluorescein (free acid), triethylamine (99.7%), xanthan gum, sodium benzoate (99%), sorbitol (99%), and glycerol were obtained from Sigma-Aldrich (UK). (3-Acryloxypropyl)trimethoxysilane (96%) was obtained from Gelest (Morrisville, USA). Ammonium solution (S.G. 0.88, 35%) was obtained from Fisher Scientific. Aerosil R972 Pharma was obtained from Laurence Industries (UK).

### Methods

#### Synthesis of Chitosan-Coated Silica Particles

Low-molecular-weight chitosan (1 g) was added to a 3% w/v aqueous solution of acetic acid (60 mL) and mixed for 30 min. The pH of the chitosan solution was adjusted to 5.5–6 using 1 M NaOH. Aerosil R972 silica (1 g) was first dispersed in ethanol (20 mL) and mixed for 15 min before addition to the chitosan solution and further mixing for 30 min. The resulting dispersion was then homogenized using a Silverson shear mixer with homogenizer attachment for five homogenization cycles of 10 min each. The resulting suspension was spray-dried at a feed rate of 3 mL·min^–1^ in a Büchi B290 spray-dryer. The inlet temperature was set at 120 ± 2 °C and the outlet temperature at 67 ± 3 °C.

#### Synthesis of Silica Particles from TEOS

Ammonium hydroxide (0.13 mL) was added to water (25 mL) and ethanol (25 mL) and mixed in an ultrasonication bath. TEOS (1 mL) was slowly added dropwise to the stirring solution. The mixture was gently stirred for 1 h before leaving to stand overnight. The particles were washed and obtained by several centrifugation and re-dispersion cycles first with ethanol, followed by a water/ethanol mixture (1:1), and finally with water. The particles were then dispersed in water and sonicated for 4 h before being freeze dried.

#### Synthesis of Acryloylated, Thiolated, and Glycidylated Silica Particles

Ammonium hydroxide (0.13 mL) was added to water (25 mL) and ethanol (25 mL) and mixed in an ultrasonication bath. TEOS (1 mL) and either GOPS, MPTS, or APTS (3.44 mL) was mixed and slowly added dropwise to the stirring solution. The dispersion was then gently stirred for an hour before leaving to stand overnight. The particles were washed and obtained by several centrifugation and re-dispersion cycles first with ethanol, followed by a water/ethanol mixture (1:1) and finally with water. The particles were then dispersed in water and sonicated for 4 h before being freeze-dried.

#### Synthesis of Phenylboronic Acid-Functionalized Silica

Glycidylated silica prepared by co-condensation of TEOS with GOPS (0.5 g) were dispersed in ethanol (30 mL). APBA (0.25 g) and TEA (0.17 mL) were added, and the mixture was refluxed for 6 h under gentle stirring. The sample was then centrifuged at 3000 rpm for 30 min and re-dispersed in ethanol several times. The remaining wet solid was dried at 50 °C under vacuum.

#### Preparation of Toothpastes

The preparation of toothpastes was adapted from our previous study.^3^ Sorbitol (50 g) and sodium benzoate (1 g) were added to water (62.5 mL) and stirred using an overhead mixer. Sodium hydroxide (0.3 g) was added during mixing and mixed until fully dissolved. Xanthan gum (2.4 g) was added to glycerol (20 g) and was premixed before adding to the sorbitol solution where it was stirred for 1 h. The polymer gel produced was used as a base to make the different toothpastes. Different types of silica particles (0.58 g) were added to the polymer gel base (2.5 g) and was mixed with a Silverson Industrial High Shear Mixer (UK) at a rate of 2000 rpm until a paste formed.

#### Characterization

Fourier-transform infrared spectroscopy (FTIR) analysis of dry silica particles was performed on a PerkinElmer Spectrum 100 FTIR measuring the region of 4000–650 cm^–1^ with a resolution of 4 cm^–1^ by accumulating 32 scans. TGA was carried out on a QSeries Q50 TGA under a nitrogen atmosphere at a heating rate of 10 °C per minute from room temperature to 700 °C. SEM was performed on a FEI Quanta FEG 600 operated at 20 kV. The particles were coated with gold prior to SEM experiments. The particle size was measured using image analysis with ImageJ software. The number of unique particles measured on each image was 100. TEM was performed on a JEM 2100 plus operated at 200 kV. The particles were dispersed in ethanol (0.05 g/L) before dropping the dispersion onto the carbon–copper grid and allowing it to dry before analysis.

#### *Ex Vivo* Wash-Off Test

*Ex vivo* wash-off tests were performed following a procedure used in our previous toothpaste retention study.^3^ Wash-off tests measuring how much saliva is required to reduce the fluorescence observed were performed to test the retentive properties of the toothpaste formulations. Background photos were taken before toothpaste application with a Leica MZ 10 F fluorescence microscope (Germany). The microscope settings were standardized throughout all the experiments: exposure time 57 ms, gain 10x, gamma 1, pseudocolor 527 nm, and intensity 3. The toothpaste was physically loaded with fluorescein-free acid (0.1 g) by mixing the paste with the fluorescent dye powder until evenly distributed. This was checked using the fluorescence microscope. The toothpaste was brushed onto the dorsum section of a sheep’s tongue cut into 3 cm^2^ squares. The tongue was photographed, and artificial saliva containing 10 mM potassium chloride, 4 mM calcium chloride, 2 mM sodium bicarbonate, 6.7 mM potassium dihydrogen phosphate, and 7 mM sodium chloride prepared in deionized water was dripped at 1 mL/min onto the tongue to mimic the natural flow of simulated saliva. Fluorescence images were taken at washes with 1, 3, 5, 7, and 10 mL of simulated saliva and analyzed using ImageJ software to measure the fluorescence intensity. Freshly excised sheep tongues were received from Newman’s Abattoir (Farnborough, UK), transported to the laboratories in an ice box and used within 24 h.

#### Statistical Analysis

The statistical analysis for all the experiments was performed using two-way ANOVA with Bonferroni corrections used on pairwise analysis to account for multiple comparisons at a significance level of *p* ≤ 0.05 on Prism (Graphpad Prism software, USA).

## Results and Discussion

**Figure 1:**
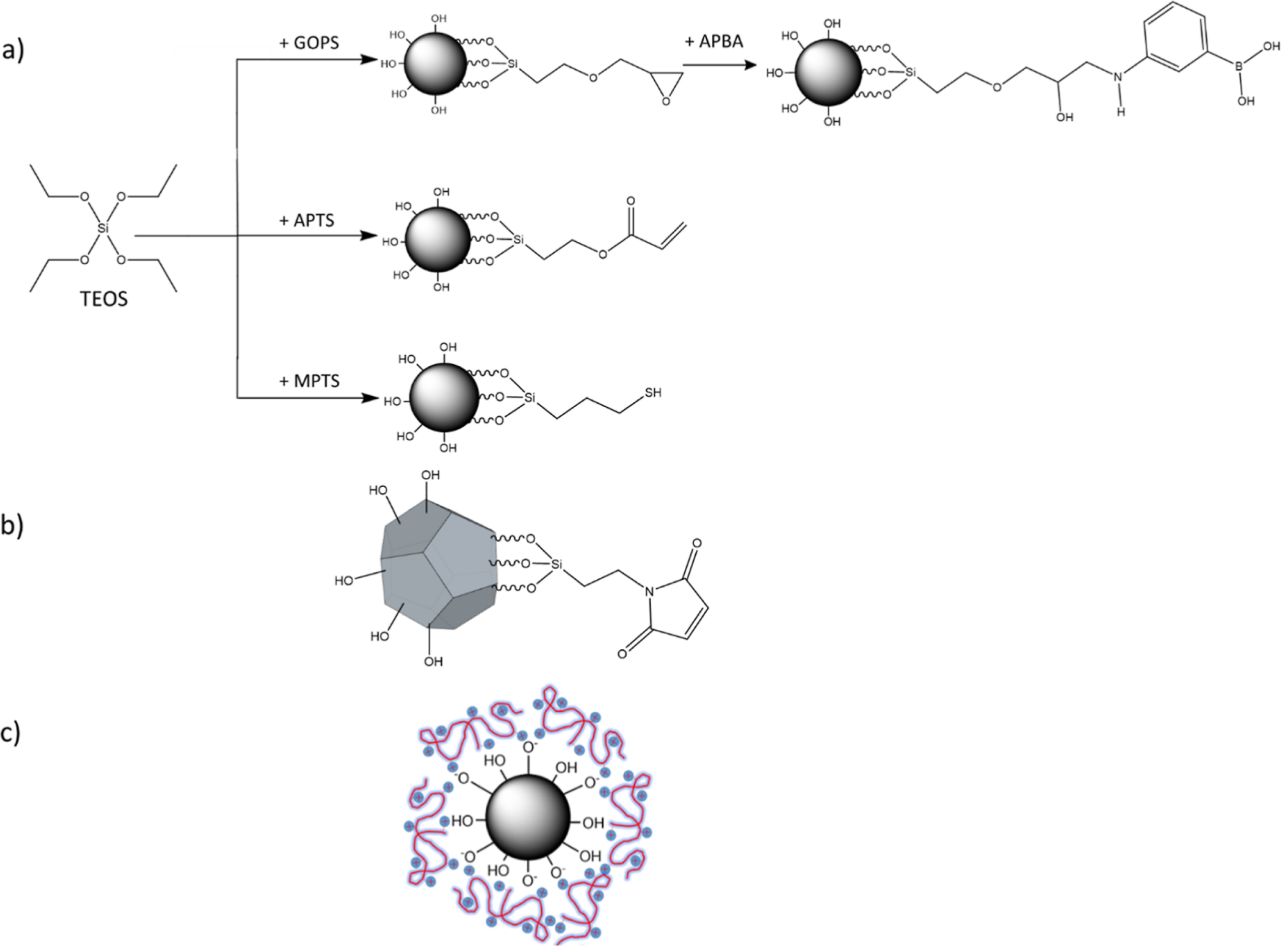
Co-condensation reactions of TEOS with GOPS, APTS, and MPTS, with subsequent modification of glycidylated silica with APBA (a); (3-maleimido)propyl-functionalized silica gel (b); and chitosan-coated Aerosil R972 (c).

Silica particles were prepared through the co-condensation of TEOS with MPTS, APTS, and GOPS. The particles synthesized with GOPS were then modified with APBA. Additionally, Aerosil R972 silica particles were coated with chitosan. These synthesized particles were compared with commercially obtained (3-maleimido)propyl-functionalized silica and silica particles synthesized from pure TEOS. The schemes of synthesis and structure of all mucoadhesive particles used in this study are shown in Figure 1.

All these types of silica particles were developed with reasonable yields, sufficient for their incorporation into the model toothpastes to evaluate the retention in the mouth. These particles were extensively characterized using FTIR spectroscopy, thermal analysis, and electron microscopy techniques.

### FTIR Spectroscopy Analysis

**Figure 2:**
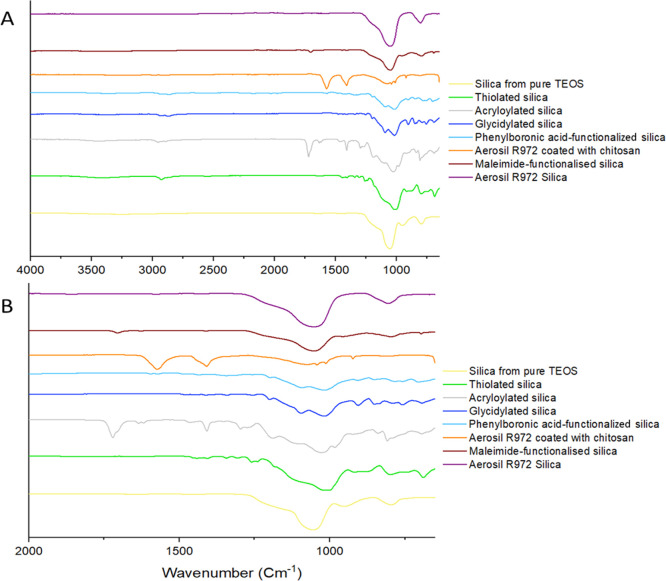
FTIR spectra of silica particles in the range of 4000–650 cm ^–1^ (A) and in the range of 2000–650 cm ^–1^ (B). The individual spectra with specific bands labeled can be found in Supporting Information (Figures S2–S5).

The prepared and commercial silica particles were analyzed using FTIR to confirm successful surface functionalization with silane compounds and chitosan (Figure 2). The spectrum of the silica nanoparticles synthesized using TEOS only showed peaks at 1055 and 795 cm^–1^ which are attributed to the stretching of Si–O groups with absorbance at 3250 cm^–1^ from hydroxyl groups present on the surface.

The structure of glycidylated silica particles was confirmed by the appearance of strong bands at 2932 and 2869 cm^–1^ corresponding to the CH_2_ stretching vibration of the methyl or methylene groups and the peak at 905 cm^–1^ corresponding to the epoxy ring. The presence of glycidyl groups on the surface of these particles then allows their chemical modification with APBA *via* the reaction of epoxy groups with amino group forming particles with mucoadhesive properties. This successful functionalization was confirmed by the appearance of peaks at 2935 and 2866 cm^–1^ attributed to the stretching of CH_2_ and the peaks at 1595, 1568, 1484, 1443, and 692 cm^–1^ being assigned to the benzene ring of the phenylboronic acid. The peak at 1342 cm^–1^ is characteristic of B–O stretching, indicating that APBA was successfully reacted with GOPS.^36^

The successful synthesis of thiolated silica was confirmed by the appearance of the band at 2555 cm^–1^ assigned to the S–H stretching. The bands at 2929 and 2886 cm^–1^ are assigned to the CH_2_ stretching from MPTS.

The structure of acryloylated silica particles was confirmed by the appearance of peaks at 2953 and 2895 cm^–1^ which are attributed to the stretching of CH_2_, the large peak at 1720 cm^–1^ attributed to the C=O of the acryloyl group, and the broad band at 3427 cm^–1^ due to hydroxyl groups present on the silica surface. Additionally, the peaks at 1635 cm^–1^ were attributed to the C=C stretching and the large peak at 1408 cm^–1^ to C–H bending.

The FTIR spectra of Aerosil R972 before modification with chitosan showed a peak at 1052 cm^–1^ assigned to the asymmetric stretching vibrations of Si–O–Si and peak at 806 cm^–1^ responsible for the symmetric deformation of the Si–O–Si bonds. Afterward, the Aerosil R972 silica modified with chitosan showed a broad peak at 3369 cm^–1^ corresponding to the stretching vibrations of OH groups with NH overlap.^37^ The peak at 2978 cm^–1^ is attributed to the stretching of C–H. The characteristic bands of chitosan assigned to the stretching vibration of the amino groups and vibration of the C–H group were present at 1571 and 1335 cm^–1^, respectively, with C–O–C stretching vibration observed at 1073 cm^–1^.^38^

The FTIR spectra of the (3-maleimido)propyl-functionalized silica gel showed peaks at 1706 cm^–1^ corresponding to the carbonyl group stretching and 799 cm^–1^ assigned to C=C bending. The characteristic Si–O stretching peak was also observed at 1053 cm^–1^.

### Thermal Gravimetric Analysis

TGA was performed on all the samples to determine their organic content. As shown in Figure S1, silica particles derived from pure TEOS showed minimal weight loss resulting only from the physically absorbed water, indicating that no impurities or residual starting material was present. The Aerosil R972 particles coated with chitosan and acryloylated silica particles showed a 47 and 52% mass loss, respectively. The particles prepared by co-condensation of GOPS and TEOS showed 48% weight loss, and when these were additionally modified with APBA, the weight loss was around 55%. The particles prepared by co-condensation of MPTS with TEOS only showed a 40% mass loss, indicating lower incorporation of thiol-containing silane into their structure.

The maleimide-functionalized silica gel only had a weight loss of 13%, indicating that there was a relatively lower level of organic moieties in this commercial sample, possibly mostly present on its surface. This could be due to the method in which the silica was functionalized, leading to a reduced grafting yield, or could be due to the type of silica used.

The grafting density of the APBA, GOPS, GOPS-APBA, and MPTS particles was determined by TGA and was calculated using eq 1
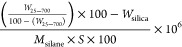
1where *W*_25___700_ is the mass loss from the degradation of the silane between 25 and 700 °C, *W*_silica_ is the weight loss of the unmodified silica (*W*_silica_ = 6.24%), *M*_silane_ is the molar mass of the silane, and *S* is the specific area of the silica.^39^

### Electron Microscopy

**Figure 3:**
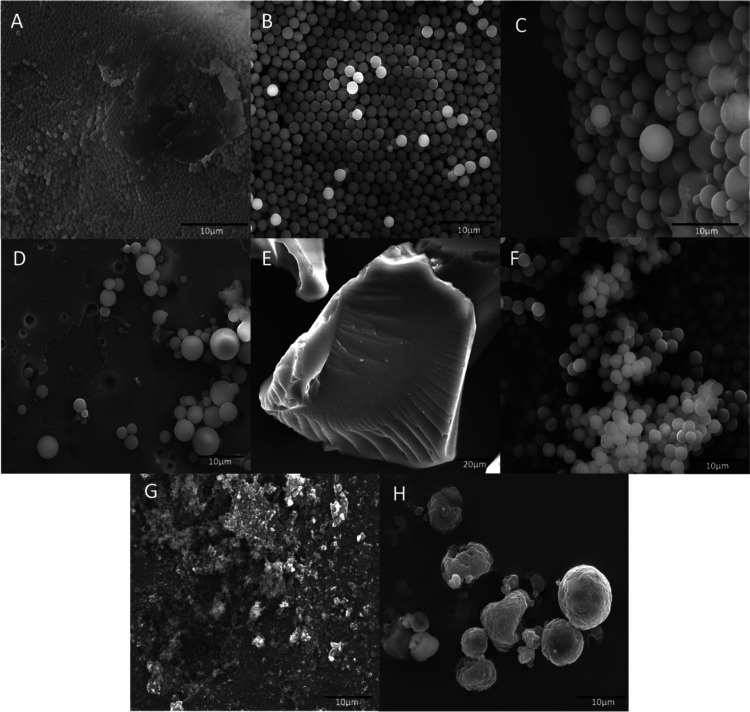
SEM images of silica derived from pure TEOS (A), acryloylated silica (B), glycidylated silica (C), silica functionalized with phenylboronic acid (D), (3-maleimido)propyl-functionalized silica gel (E), thiolated silica (F), Aerosil R972 silica (G), and Aerosil R972 silica coated with chitosan post spray-drying (H).

The morphology of all the silica particles used in this work was investigated by SEM (Figure 3).

The silica derived from pure TEOS and APTS-TEOS mixtures possessed a smooth surface and uniform size, whereas the particles formed from GOPS-TEOS mixtures as well as the product of their modification with APBA varied in size but still had a smooth spherical surface. The (3-maleimido)propyl-functionalized particles unsurprisingly had the largest average size and uneven shape given that it was made from silica gel. The particles had a grain-like structure with smooth surfaces much larger than the silica synthesized in this work. As these samples were functionalized on a different type of silica to the one used in the synthesis of the other particles, this was expected. The thiolated silica formed tight groups of spherical particles possibly due to some disulfide bridges forming between them. The average particle size was determined through the analysis of SEM images taken from multiple samples (Table 2). An increase in the particle size was seen on the modification of glycidylated particles with APBA and on coating of Aerosil R972 silica with chitosan, which was expected with the formation of an extra functional layer to their surfaces. Aerosil R972 coated with chitosan also formed spherical particles which were not present in the unmodified particles, but these were not fully uniform in size.

**Table 1:** Grafting Density Determined from TGA of Silica Particles

sample	grafting density (μmol·m^–2^)
acryloylated silica	0.53
glycidylated silica	0.53
phenylboronic acid-functionalized silica	0.44
thiolated silica	0.45

**Table 2:** Particle Size Calculated from SEM Image Analysis of Silica Samples^a^

sample	average particle size (μm)
silica derived from pure TEOS	0.5 ± 0.0
acryloylated silica	1.8 ± 0.2
glycidylated silica	3.2 ± 1.2
phenylboronic acid-functionalized silica	3.3 ± 1.2
thiolated silica	2.3 ± 0.1
(3-maleimido)propyl-functionalized silica	78.7 ± 8.4
Aerosil R972 coated with chitosan	8.9 ± 3.0

a To determine the average particle size, the number of unique particles for each sample measured was 100.

**Figure 4:**
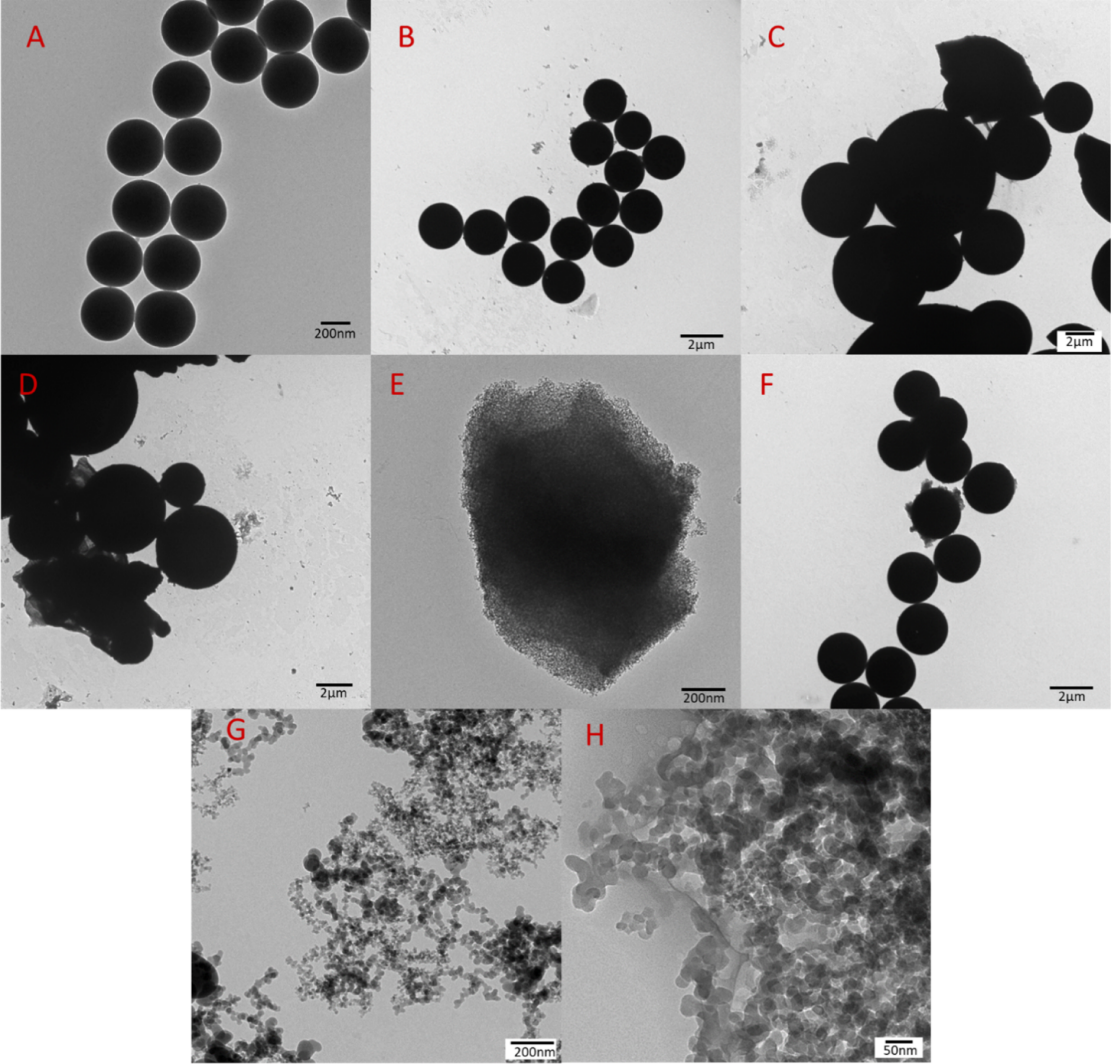
TEM images of silica derived from pure TEOS (A), acryloylated silica (B), glycidylated silica (C), silica functionalized with phenylboronic acid (D), (3-maleimido)propyl-functionalized silica gel (E), thiolated silica (F), Aerosil R972 silica (G), and Aerosil R972 silica coated with chitosan (H).

The TEM images further confirmed that all the synthesized and modified silica particles were spherical in nature with TEOS, acryloylated, and thiolated silica, forming uniform particles of comparable size to each other, respectively. The GOPS and GOPS-APBA particles, however, varied in size, as seen in Figures 3 and 4C,D, but their average size overall was very similar with the phenylboronic acid-functionalized particles having a larger average size than the glycidylated silica (Table 1). The TEM images of the particles further confirmed the trends seen in the SEM images (Figure 4). The TEM image of the (3-maleimido)propyl-functionalized silica gel further showed its porous nature which was not observable in the SEM image. The chitosan-coated Aerosil R972 silica exhibited good transparency, suggesting that the nanoparticles did not aggregate, and in turn due to the lack of phase separation in the polymeric matrix, they can be considered as a nanocomposite of homogeneously dispersed reinforcement within the chitosan matrix. This was also seen in the work carried out by da Costa Neto *et al.*(37)

Functionalized silica particles have previously been reported as mucoadhesive carriers in a limited number of studies in drug delivery.^11^ However, to the best of our knowledge, there are no studies in the literature that report the addition of functionalized silica to toothpastes to improve their retention in the mouth. One issue that may arise from the addition of the silica to toothpaste formulations is an alteration in the product properties. Work done by Honary *et al.*(40) found that the size of particles plays a role in the spreadability of the pharmaceutical and cosmetic pastes. However, as the functionalized silica incorporated into the toothpastes were a replacement for the particles with comparable sizes which would normally be used in these formulations, the addition of the silica should not affect their rheological and flow properties.^10^

### Toothpaste Wash-Off Test

**Figure 5:**
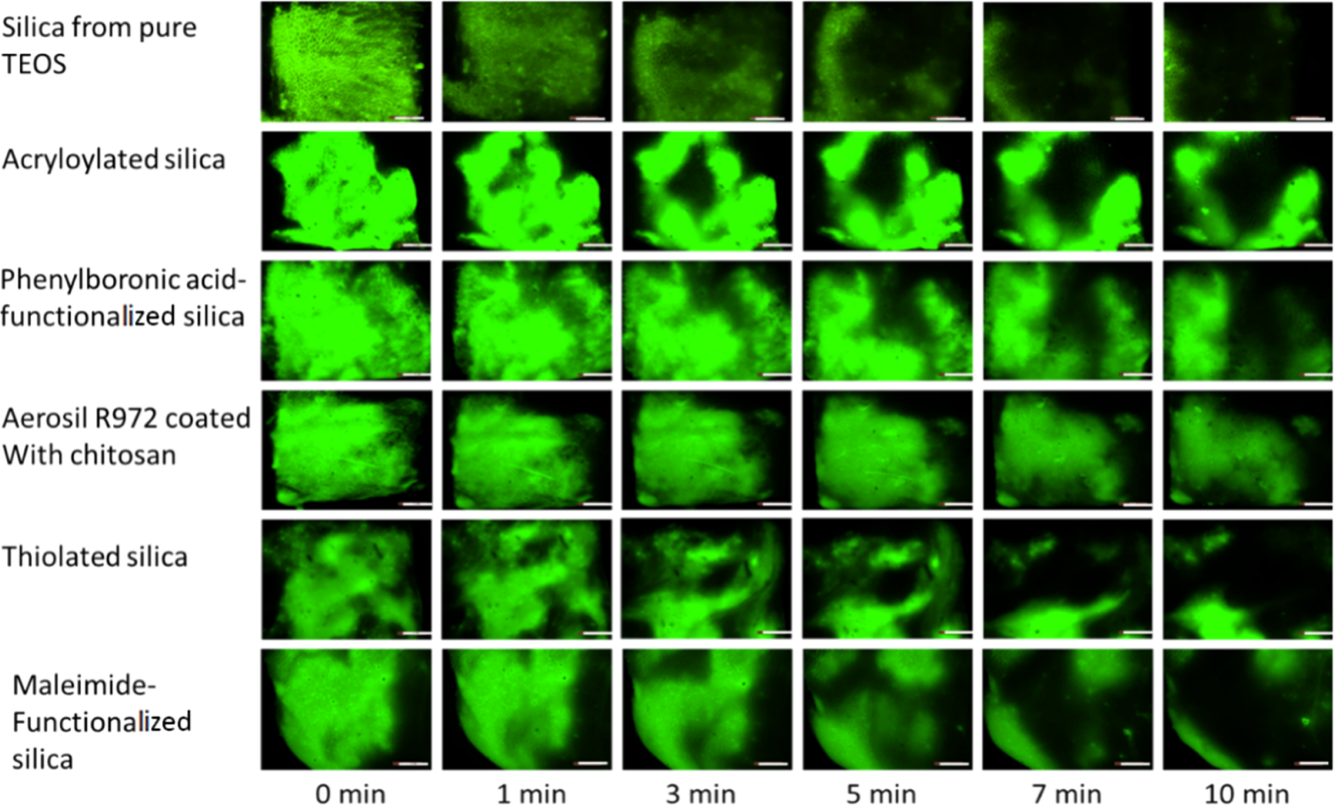
Fluorescence images of the tongue after being brushed with fluorescently labeled toothpaste made with unmodified and functionalized silica and subsequently washed with different volumes of artificial saliva. Scale bar 5 mm.

Wash-off tests were performed to measure the retentive properties of the functionalized silica formulated into toothpaste compared to a control formulation made using silica derived from pure TEOS (Figure 5). This technique has previously been used by our research group to examine the role of mucoadhesives on toothpaste retention in the oral cavity.^9^ The fluorescence images allow the observation and monitoring of toothpaste and its movement during wash-off tests and the measurement of the fluorescence intensity and its decrease over the course of a test with higher intensities, indicating an increase in the retention of the said formulation.

The oral cavity poses a lot of issues that must be overcome to ensure that a therapeutic dose is delivered to the active sites. The vascular nature of the oral cavity combined with mastication, dissolution of active ingredients in saliva, and finally swallowing food quickly remove active ingredients, flavors, and any residue left behind post brushing, decreasing the effectiveness of the formulation.^4,41^ In addition, the use of mouthwash post brushing also reduces the effectiveness of the formulations by removing fluoride compounds, antibacterial agents, and other active ingredients.^41^

All the functionalized silica formulations showed retentive properties significantly greater than the unmodified control derived from pure TEOS. The fluorescence intensity of the toothpaste made with unmodified silica can be seen decreasing more rapidly than the modified silica formulations as seen in Figure 5.

**Figure 6:**
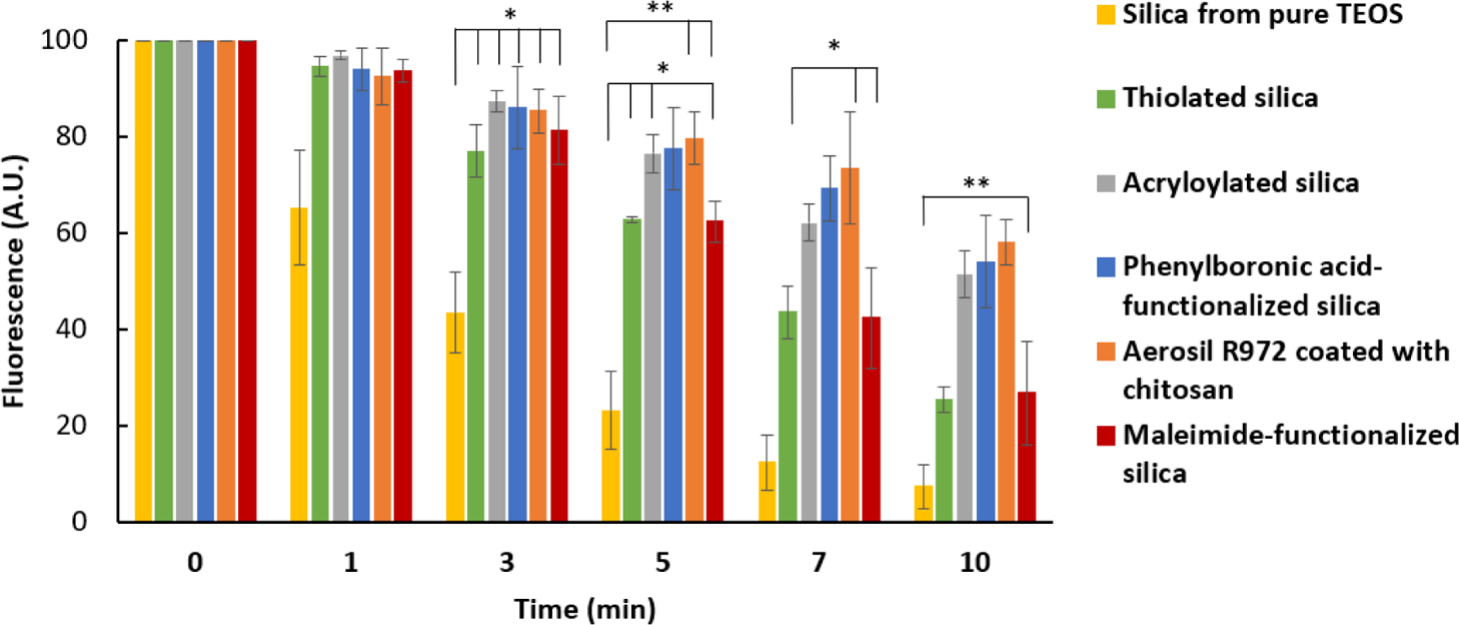
Fluorescence levels observed for toothpaste formulations made with functionalized silica after being applied to the tongue and washed with artificial saliva. Error bars represent standard deviation. All tests were performed in triplicate.

The fluorescence intensity of the toothpaste prepared with silica functionalized with mucoadhesive groups or polymer (chitosan) after washing with artificial saliva does not decrease as rapidly as the control, indicating a higher retention of the formulation on the mucosal surface. This result shows that the incorporation of the modified silica improves the retentive properties of the formulation and is observable by the reduced decrease in fluorescence of the samples compared to the control. The fluorescence images were processed using image analysis, and results are presented in Figure 6.

The toothpastes with all types of functionalized silica particles showed greater retention of fluorescein on the mucosal surface. The toothpastes containing the particles coated with chitosan, as well as the silica with acryloyl- and phenylboronic groups, showed the best results and similar levels of fluorescein retention throughout the test. This is observable in the images where the three mentioned samples show only a slight decrease in fluorescence throughout the test. Although the mucosal retention of the fluorescein in the toothpastes containing thiolated particles and the (3-maleimido)propyl-functionalized silica was lower than that of the acryloyl-, phenylboronic acid-, and chitosan-functionalized silica, they still exhibited better performance compared to the control formulation. Perhaps, poorer retention of the maleimide-functionalized silica was related to the lower levels of maleimide groups as was established in the thermal analysis experiments. Similarly, lower levels of thiol groups were also observed in thiolated silica. Another possible limitation of maleimide-functionalized silica to facilitate better retention could be related to its substantially larger particle size that will inhibit its deeper penetration and deposition on the mucosal surface.

The improved retention of toothpastes with functionalized silica is due to the enhanced mucoadhesive properties of these particles. However, the mechanisms of mucoadhesion enhancement are different for each type of particles. Silica particles coated with chitosan adhere to mucosal surfaces predominantly due to electrostatic interactions,^32^ whereas other functionalized particles are capable of forming covalent linkages with some functional groups present in mucins. Thiolated, acryloylated, and maleimide-functionalized silica form covalent bonds with thiol groups present in cysteine-rich domains of mucin, whereas phenylboronic acid-functionalized silica forms dynamic covalent bonds with 1,2-cis-diols present in the oligosaccharide fragments of mucins.^20^

## Conclusions

Silica particles functionalized with mucoadhesive groups (thiol, acryloyl, and phenylboronic acid) or coated with mucoadhesive polymer (chitosan) were successfully synthesized and characterized. Additionally, a commercially available sample of maleimide-functionalized silica was used. All these particles were used to formulate model toothpastes, and their *ex vivo* retention on freshly excised sheep tongues was evaluated using a fluorescence microscopy-based flow-through assay. The sample producing functionalized silica was shown to improve the retention of toothpaste when incorporated into the formulation and retained on the mucosal tissue for a significantly longer time compared to the unmodified control formulation. The results from this work indicate that replacing the silica used in current commercial pastes with functionalized variants, specifically with those with chitosan or functionalized with acryloyl and phenylboronic acid groups, provided a longer retention time of the whole formulation on oral mucosal surfaces. This work lays the foundation for incorporating modified silica particles into other semisolid formulations as a method for improving their mucoadhesive properties.

## Supporting Information Available

Data of TGA and FTIR spectra (PDF)
