# Supplementary material for: Surface Modification of Silica Particles with Adhesive Functional Groups or Their Coating with Chitosan to Improve the Retention of Toothpastes in the Mouth
Source: Langmuir. 2023 Jan 17;39(4):1677–85. doi: 10.1021/acs.langmuir.2c03269 (PMC9893808; doi:10.1021/acs.langmuir.2c03269)
Supplement: Supplementary file 1 — la2c03269_si_001.pdf [file la2c03269_si_001.pdf]

Supporting Information

Surface modification of silica particles with adhesive functional groups or their coating with chitosan to improve retention of toothpastes in the mouth

*Sam R. Aspinall [a] and Vitaliy V. Khutoryanskiy [b]**

a Department of Pharmacy & Research Centre in Topical Drug Delivery and Toxicology, University of Hertfordshire, Hatfield, AL10 9AB Hertfordshire, UK

b Department of Pharmacy, University of Reading, Whiteknights, PO Box 224, Reading RG6 6DX, UK

Table of contents:

Figure S1. TGA curves of unmodified and functionalised silica particles.

Figure S2. FTIR spectra of silica particles and glycidylated silica & phenylboronic acid-functionalised silica particles in the range of 3000-650 cm -1 with key peaks labelled as discussed in the FT-IR spectroscopy analysis.

Figure S3. FTIR spectra of silica particles and thiolated silica particles in the range of 3500-650 cm^{-1} with key peaks labelled as discussed in the FTIR spectroscopy analysis.

Figure S4. FTIR spectra of silica particles and acryloylated silica particles in the range of 4000-650 cm-1 with key peaks labelled as discussed in the FTIR spectroscopy analysis.

Figure S5. FTIR spectra of Aerosil R972 silica and Aerosil R972 coated with chitosan in the range of 4000-650 cm^{-1} with key peaks labelled as discussed in the FT-IR spectroscopy analysis.

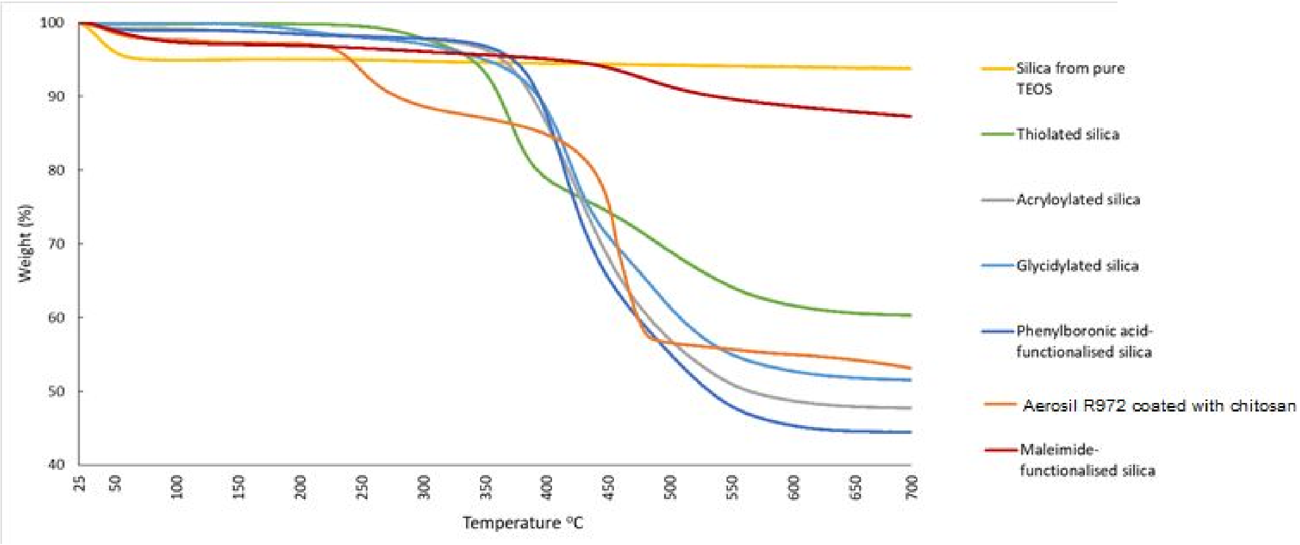

Figure S1. TGA curves of unmodified and functionalised silica particles.

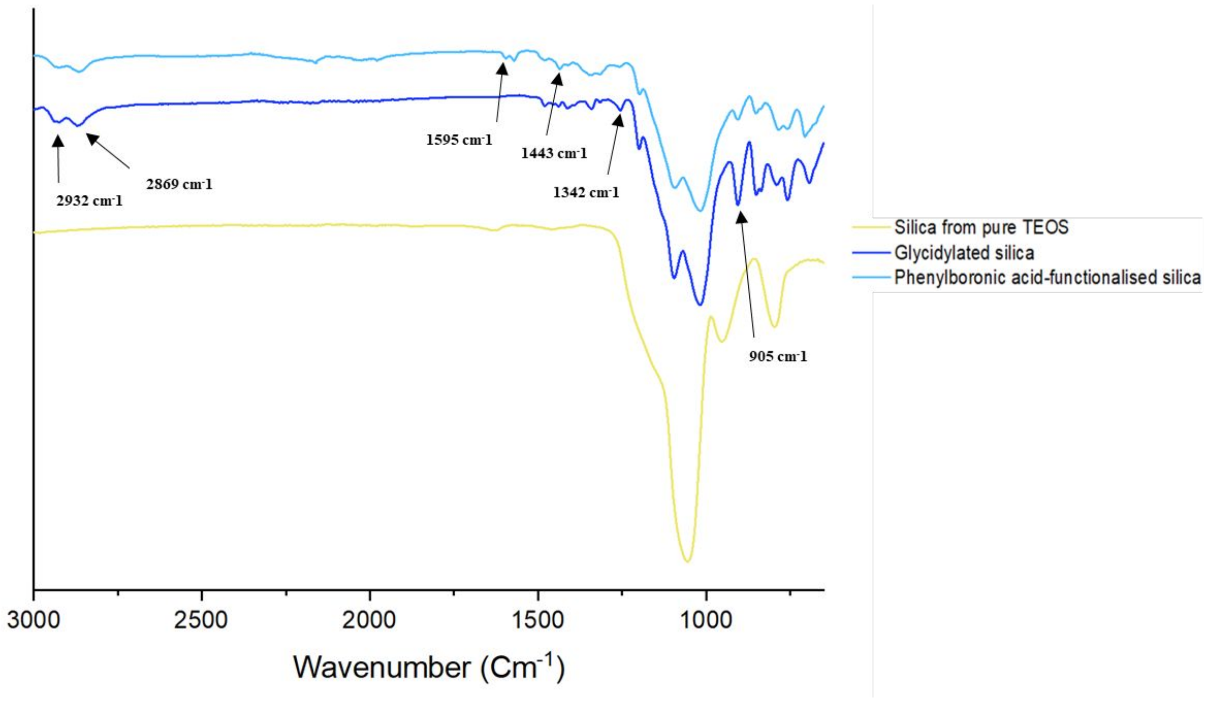

Figure S2. FTIR spectra of silica particles and glycidylated silica & phenylboronic acid-functionalised silica particles in the range of 3000-650 cm $^{-1}$ with key peaks labelled as discussed in the FT-IR spectroscopy analysis.

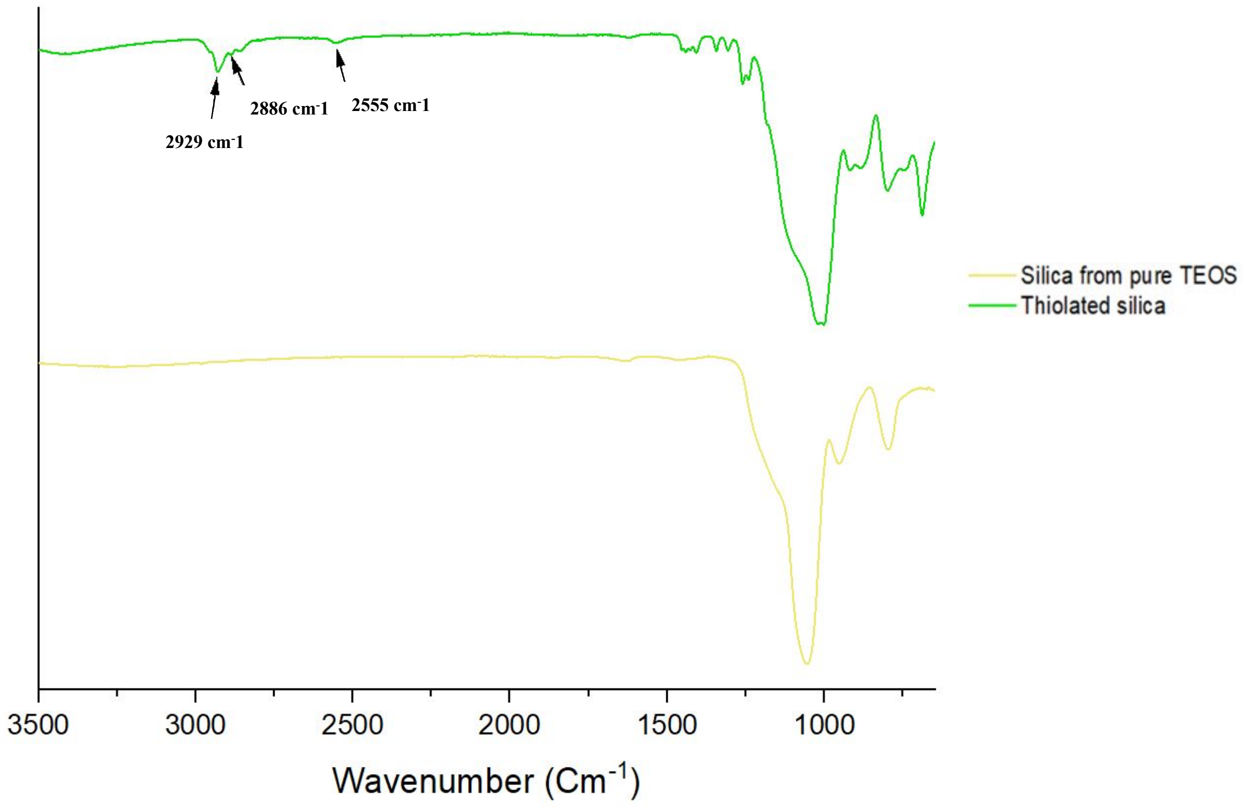

Figure S3. FTIR spectra of silica particles and thiolated silica particles in the range of 3500-650 cm^{-1} with key peaks labelled as discussed in the FTIR spectroscopy analysis.

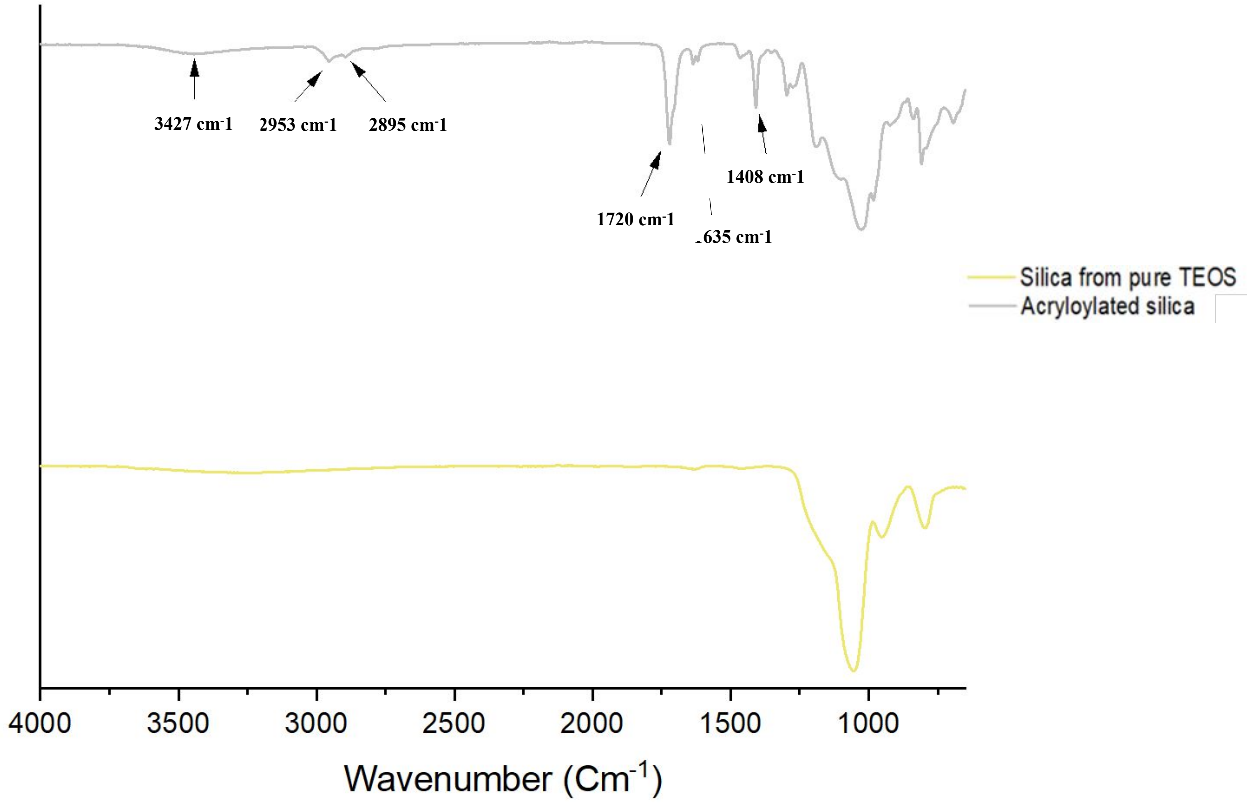

Figure S4. FTIR spectra of silica particles and acryloylated silica particles in the range of 4000-650 cm^{-1} with key peaks labelled as discussed in the FTIR spectroscopy analysis.

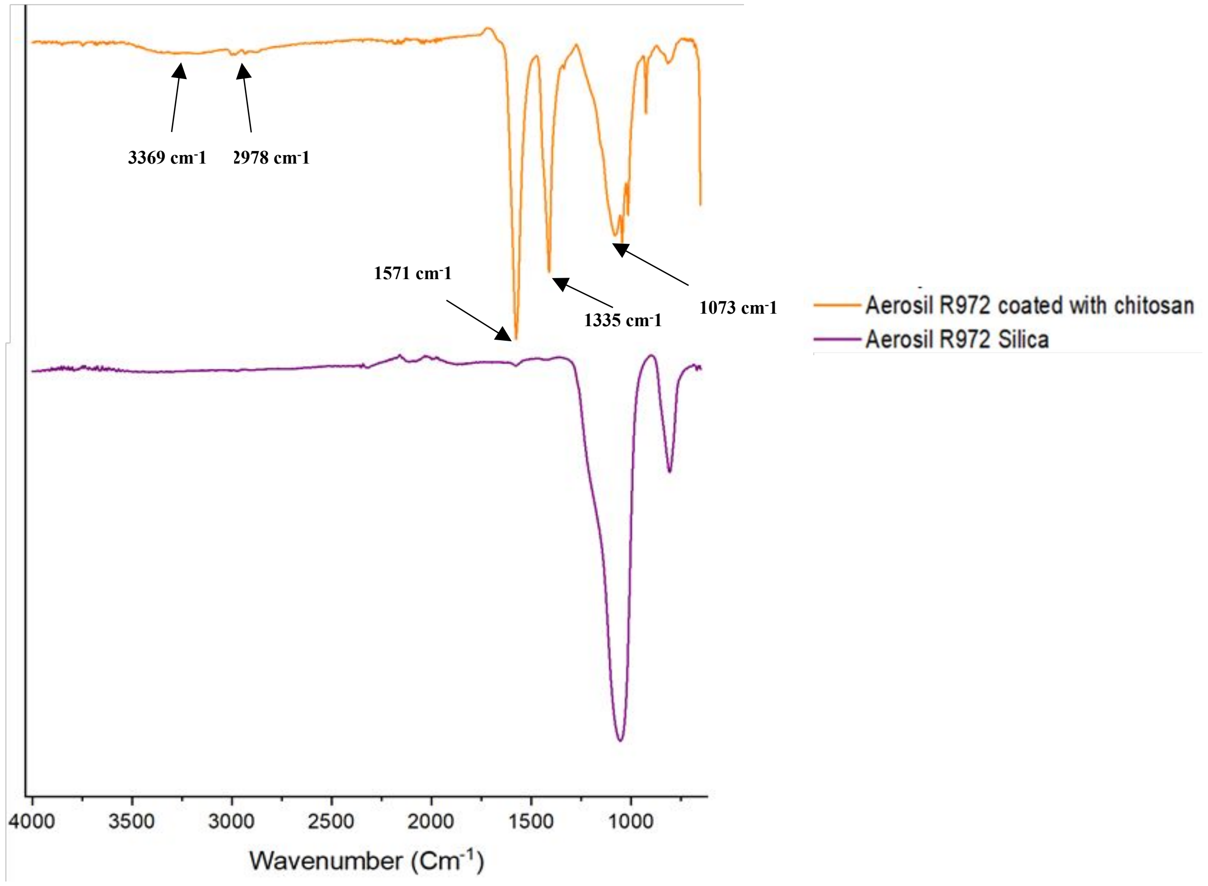

Figure S5. FTIR spectra of Aerosil R972 silica and Aerosil R972 coated with chitosan in the range of 4000-650 cm^{-1} with key peaks labelled as discussed in the FT-IR spectroscopy analysis.
